# Microwave fabrication of Cu_2_ZnSnS_4_ nanoparticle and its visible light photocatalytic properties

**DOI:** 10.1186/1556-276X-9-477

**Published:** 2014-09-09

**Authors:** Zhihua Zhou, Pingan Zhang, Yuelai Lin, Eric Ashalley, Haining Ji, Jiang Wu, Handong Li, Zhiming Wang

**Affiliations:** 1 State Key Laboratory of Electronic Thin Films and Integrated Devices, School of Microelectronics and Solid-State Electronics, University of Electronic Science and Technology of China, Chengdu 610054, P. R. China

**Keywords:** Cu_2_ZnSnS_4_, Microwave fabrication, Photocatalyst

## Abstract

Cu_2_ZnSnS_4_ nanoparticle with an average diameter of approximately 31 nm has been successfully synthesized by a time effective microwave fabrication method. The crystal structure, surface morphology, and microstructure of the Cu_2_ZnSnS_4_ nanoparticle were characterized. Moreover, the visible light photocatalytic ability of the Cu_2_ZnSnS_4_ nanoparticle toward degradation of methylene blue (MB) was also studied. About 30% of MB was degraded after 240 min irradiation when employing Cu_2_ZnSnS_4_ nanoparticle as a photocatalyst. However, almost all MB was decomposed after 90 min irradiation when introducing a small amount of H_2_O_2_ as a co-photocatalyst. The enhancement of the photocatalytic performance was attributed to the synergetic effect between the Cu_2_ZnSnS_4_ nanoparticle and H_2_O_2_. The detailed photocatalytic degradation mechanism of MB by the Cu_2_ZnSnS_4_ was further proposed.

## Background

Organic dyes widely used in textile and plastic industries are one of the chief sources of contaminants in wastewater. They have induced serious environmental problems due to their potential toxicity to living organisms. Degradation and total removal of such contaminants are keys to ensuring a protected environment. A photocatalytic technique is considered to be a promising method for treating organic dyes in wastewater [1]. However, an obvious challenge for degradation of organic dyes is that most photocatalysts, such as TiO_2_ or BiVO_4_[2,3], are only effective in the UV range. To broaden their light absorption range, various methods including dye sensitizing [4], metal doping [5] non-metal doping [6,7], and noble metal decorating [8] have been developed. However, stable and efficient dyes are rare and expensive. Moreover, dopant impurity atoms in photocatalysts often serve as recombination centers for photogenerated holes and electrons [9]. To avoid these problems, great efforts have also been put into the development of alternative undoped photocatalysts which work under visible light irradiation. Until now, many materials with attractive visible light photocatalytic performance, such as Bi_2_TiO_4_F_2_[10], Bi_2_O_3_[11], AgNbO_3_[12], and graphene oxide enwrapped Ag/AgX (X = Br, Cl) nanocomposite [13] have been investigated. However, the supply of rare elements of Ag, Bi, and Nb is a critical issue for widespread use. Thus, it is crucial to investigate alternative cost-effective visible-light-driven photocatalysts.

Cu_2_ZnSnS_4_ is a direct bandgap p-type semiconductor with a high optical absorption coefficient of about 10^5^ cm^−1^[14,15]. Its elements are environmentally friendly and abundant in the earth's crust. As its bandgap is around 1.5 eV, it can absorb most of the visible light. It has been reported that Cu_2_ZnSnS_4_ possesses high photocorrosion resistance in air and aqueous solution [16]. Both of these superior properties of Cu_2_ZnSnS_4_ enrich its potential use in solar-energy-related applications.

In this work, we have fabricated the Cu_2_ZnSnS_4_ nanoparticle by a facile microwave fabrication method. The advantages of this method are the following: it is economical of time and cost effective. The crystal structure and surface morphology of the prepared Cu_2_ZnSnS_4_ nanoparticle were characterized. Moreover, the photocatalytic performance of the Cu_2_ZnSnS_4_ nanoparticle toward the degradation of methylene blue (MB) under visible light irradiation was also investigated. The Cu_2_ZnSnS_4_ nanoparticle showed noteworthy visible light photocatalytic ability.

## Methods

The Cu_2_ZnSnS_4_ nanoparticle was synthesized by a facile microwave fabrication method. Cu(CH_3_COO)_2_ · H_2_O, Zn(CH_3_COO)_2_, Sn(CH_3_COO)_2_, and thiocarbamide with a molar ratio of 2:1:1:4 were employed as source materials. All the reagents were analytically pure and bought from Sinopharm Chemical Reagent Co., Ltd, Shanghai, China. Typically, 1.123 g of the source materials was dissolved in 20 mL ethylene glycol solution as precursor. Then the precursor was stirred gently and heated in a microwave reactor (MCR-3, Gongyi City Yuhua Instrument Co., Ltd, Gongyi City, China) at 180°C for 10 min. After the vacuum filtration and drying process, the Cu_2_ZnSnS_4_ nanoparticle sample was obtained.

The crystal structure of the Cu_2_ZnSnS_4_ nanoparticle was investigated by X-ray diffraction (XRD; D/max-2200/PC, Rigaku, Tokyo, Japan) and Raman spectroscopy (Senterra, Bruker, Billerica, USA). The surface morphology and microstructure of the Cu_2_ZnSnS_4_ were measured by scanning electron microscopy (SEM; JSM 5800LV, JEOL, Tokyo, Japan) and transmission electron microscopy (TEM; JEM-2100, JEOL, Tokyo, Japan).

The photocatalytic properties of the prepared Cu_2_ZnSnS_4_ nanoparticle were investigated by employing MB as a model dye. The Cu_2_ZnSnS_4_ nanoparticle (20 mg) was dispersed in 100 mL of MB aqueous solution (10 mg/L). Prior to irradiation, the MB solution over the catalyst was gently stirred in the dark for 30 min to reach equilibrium adsorption state. Then the solution was illuminated with a 100-W xenon light source (Shanghai Yaming Lighting Co., Ltd., Shanghai, China). The concentration change of MB was monitored by measuring UV-vis absorption of the extracted MB solution at regular intervals. The characteristic peak absorbance of MB at 665 nm was used to determine its concentration. In addition, the photocatalytic properties of the Cu_2_ZnSnS_4_ nanoparticle with the assistance of 0.1 mL H_2_O_2_ (30% aqueous solution) were further investigated in the same measurement process. For comparison, a control experiment without adding Cu_2_ZnSnS_4_ and H_2_O_2_ was also carried out.

## Results and discussion

**Figure 1:**
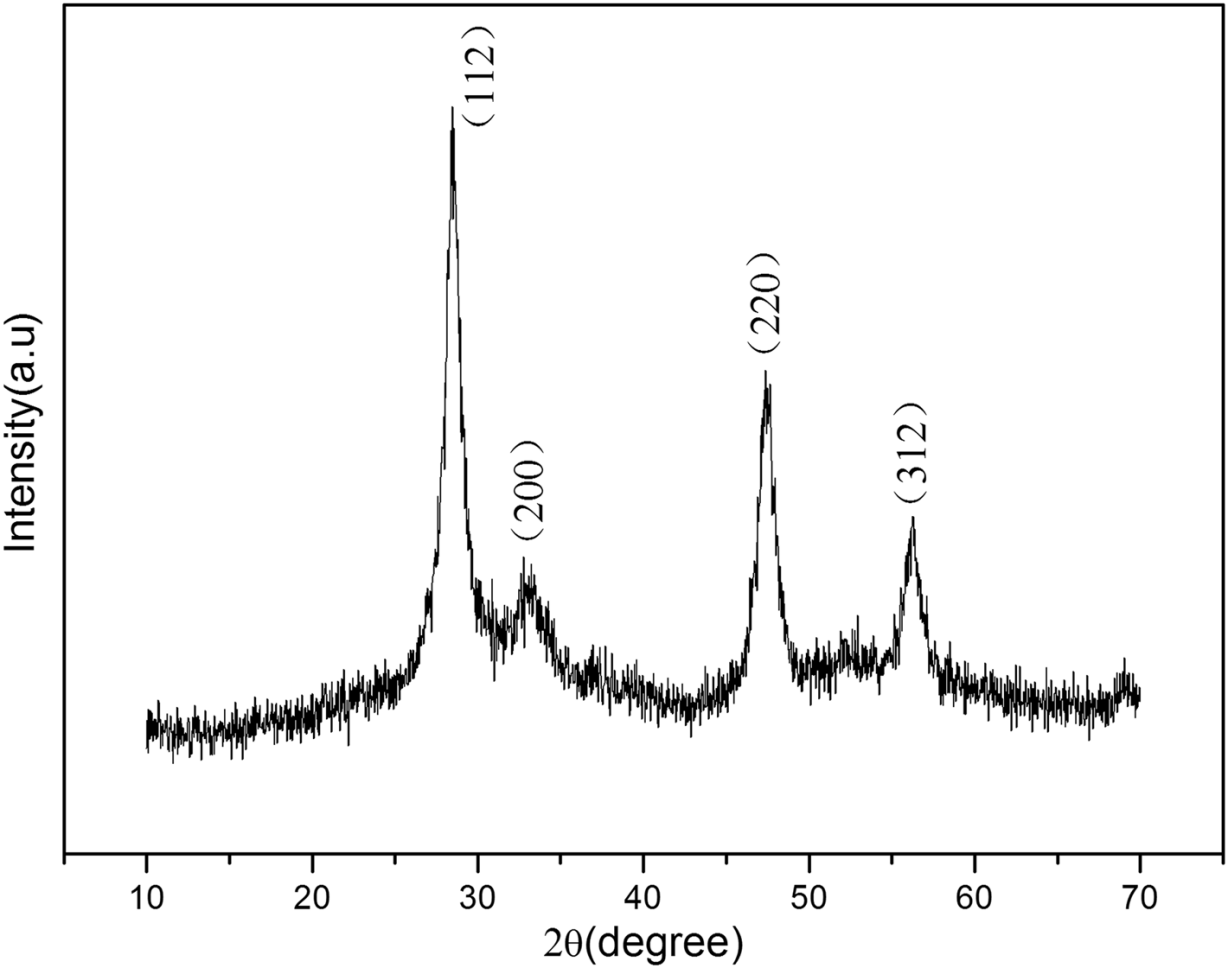
**XRD pattern for the Cu**_**2**_**ZnSnS**_**4 **_**nanoparticle.** The peaks have been indexed to kesterite Cu_2_ZnSnS_4_ (JCPDS No. 26-0575).

The crystal structure of the prepared Cu_2_ZnSnS_4_ nanoparticle is shown in Figure 1. The observed diffraction peaks at 2θ = 28.48°, 32.77°, 47.38°, and 56.26° correspond to the Cu_2_ZnSnS_4_ crystal planes (112), (200), (220), and (312), which match well with the standard XRD data file of Cu_2_ZnSnS_4_ (JCPDS No. 26-0575). No other crystalline by-products were observed in the pattern, suggesting that the as-prepared sample was pure Cu_2_ZnSnS_4_. Additionally, the strong relative intensity of the (112) and (220) lines indicates the Cu_2_ZnSnS_4_ nanoparticle is preferentially oriented in the (200) and (110) directions during the growing process.

**Figure 2:**
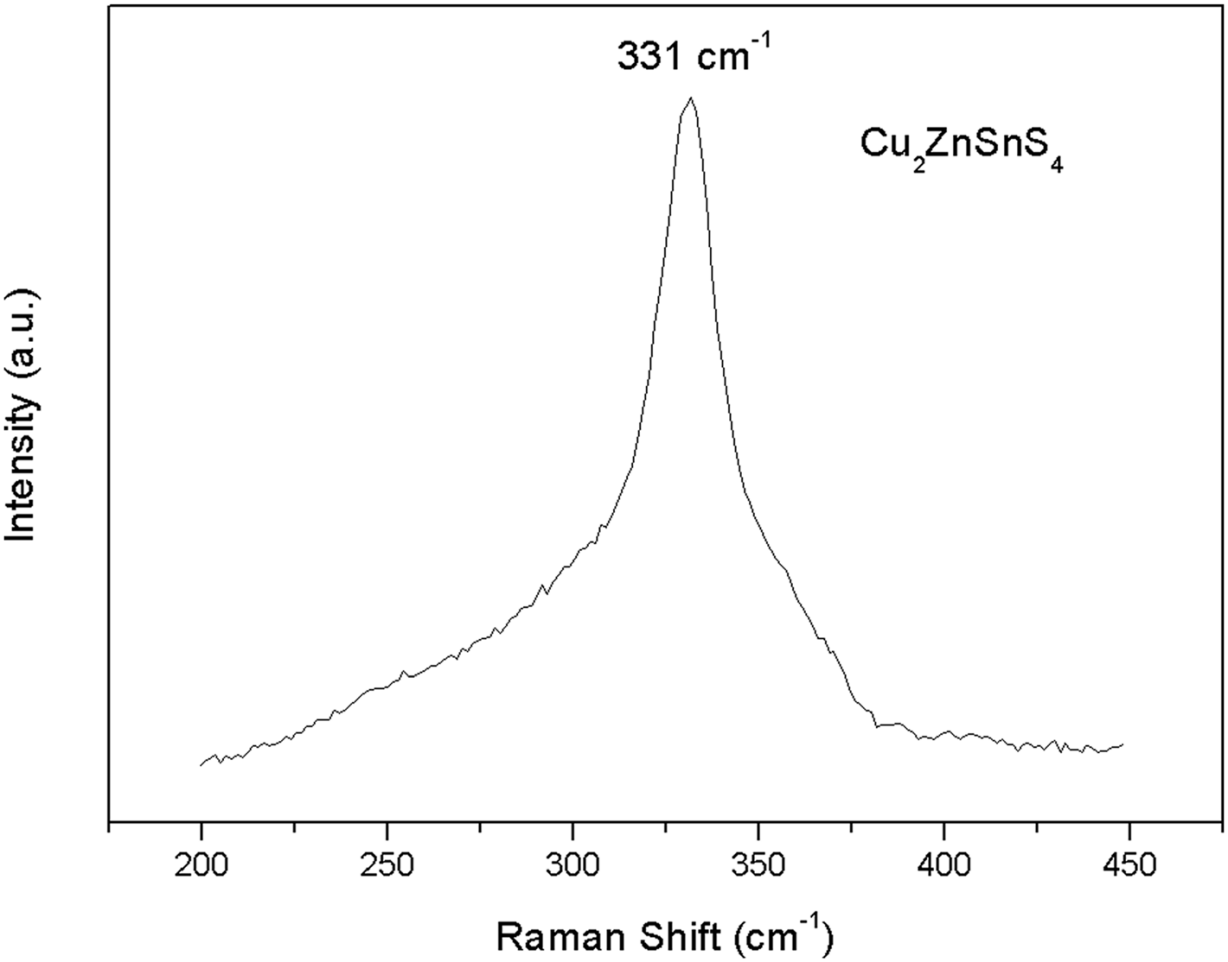
**Raman spectra of the prepared Cu**_**2**_**ZnSnS**_**4 **_**nanoparticle.**

In addition, it has been reported that the spectra of Cu_2_ZnSnS_4_ and β-ZnS are very similar in the XRD analysis results [17]. Raman spectroscopy analysis is a feasible method to distinguish Cu_2_ZnSnS_4_ from β-ZnS [18]. Therefore, we employed Raman spectroscopy to further confirm the structure of the prepared Cu_2_ZnSnS_4_ nanoparticle. A Raman spectrum of the prepared Cu_2_ZnSnS_4_ nanoparticle over the wave number range of 200 to 450 cm^−1^ is shown in Figure 2. There is an intensive peak located at approximately 331 cm^−1^, which suggests the existence of Cu_2_ZnSnS_4_[17,19]. The characteristic peaks of β-ZnS located at 348 and 356 cm^−1^ are not observed in the spectrum [20], indicating the absence of β-ZnS.

**Figure 3:**
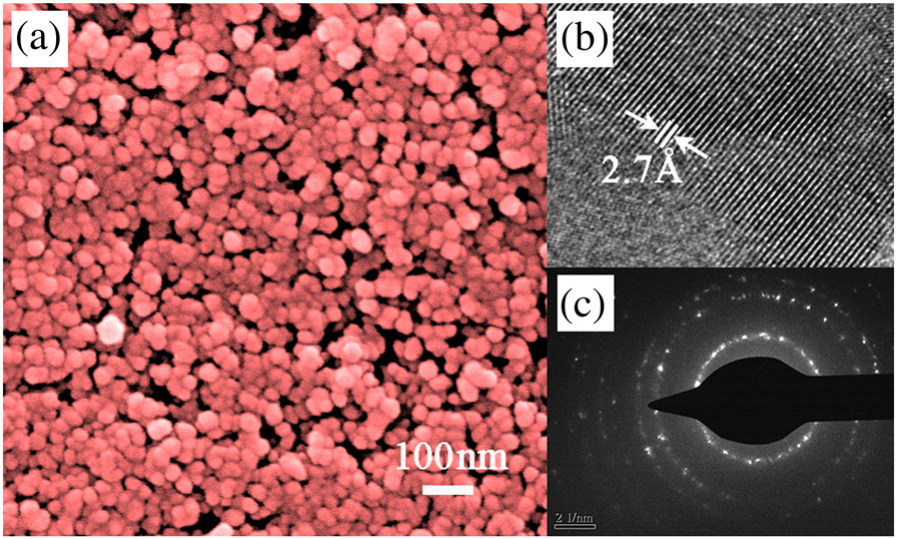
**SEM, HRTEM, and SAED images of the prepared Cu**_**2**_**ZnSnS**_**4 **_**nanoparticle. (a)** Scanning electron microscopy (SEM), **(b)** high-resolution transmission electron microscopy (HRTEM), and **(c)** selected area electron diffraction (SAED) images of the prepared Cu_2_ZnSnS_4_ nanoparticle.

Surface morphology and microstructure of inorganic semiconductor materials are of vital importance to their optoelectronic properties. Accordingly, the surface morphology of the Cu_2_ZnSnS_4_ nanoparticle was studied by SEM. Figure 3a demonstrates a representative surface morphology of the Cu_2_ZnSnS_4_ nanoparticle. It can be seen that the Cu_2_ZnSnS_4_ nanoparticle possesses similar sizes in diameter and packs uniformly. The average diameter of the Cu_2_ZnSnS_4_ nanoparticle is approximately 31 nm calculated from randomly selected 100 nanoparticles. High-resolution transmission electron microscopy (HRTEM) was further employed to investigate the microstructure of the Cu_2_ZnSnS_4_ nanoparticle. Figure 3b shows a typical HRTEM image of the Cu_2_ZnSnS_4_ nanoparticle. The interplanar spacing of 2.7 Å corresponds to the (200) plane of kesterite Cu_2_ZnSnS_4_. The selected area electron diffraction (SAED) pattern shown in Figure 3c suggests the polycrystalline nature of the nanoparticle.

**Figure 4:**
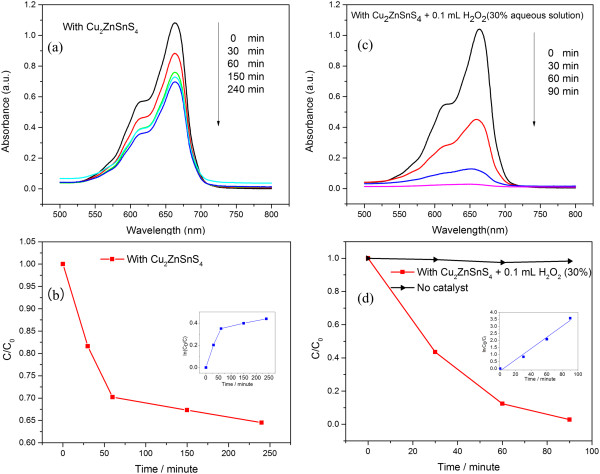
**Photocatalytic activity of the Cu**_**2**_**ZnSnS**_**4 **_**nanoparticle toward the photodegradation of methylene blue (MB). (a)** Time-dependent UV-vis absorbance spectra of the MB solution using the prepared Cu_2_ZnSnS_4_ nanoparticle as a photocatalyst. **(b)** Curves of the degradation rate of the MB dye with the prepared Cu_2_ZnSnS_4_ nanoparticle (*C*_0_ is the initial concentration of MB; *C* is the reaction concentration of MB at time *t*). The inset shows the ln(*C*_0_/*C*) versus time curve of the photodegradation of MB. **(c)** Time-dependent UV-vis absorbance spectra of the MB solution using the prepared Cu_2_ZnSnS_4_ nanoparticle and 0.1 mL H_2_O_2_ as photocatalysts. **(d)** Curves of the degradation rate of the MB dye with the prepared Cu_2_ZnSnS_4_ nanoparticle and 0.1 mL H_2_O_2_ as photocatalysts. The inset presents the ln(*C*_0_/*C*) versus time curve of the photodegradation of MB.

To evaluate the photocatalytic performance, we analyzed the decomposition of the (MB) dye in aqueous solution over the Cu_2_ZnSnS_4_ nanoparticle under visible light irradiation. Figure 4a presents the time-dependent absorption spectra of MB degradation over the Cu_2_ZnSnS_4_ nanoparticle upon visible light irradiation. The five curves in the pattern are the UV-vis spectra of MB solutions extracted at 0 min, 30 min, 60 min, 150 min, and 240 min. It can be observed that the absorbance peak at 665 nm, which is the characteristic absorption peak of MB, reduced slowly with increasing irradiation time. After 240 min, only about 30% of MB was degraded. In addition, it was difficult to further degrade MB by increasing the irradiation time. However, with the addition of 0.1 mL H_2_O_2_ (30% aqueous solution), as shown in Figure 4c, almost all MB was degraded after 90 min irradiation. In general, peroxymonosulfate, peroxydisulfate, and H_2_O_2_ are often employed to assist in evaluating the photocatalytic properties of semiconductor materials. Both peroxymonosulfate and peroxydisulfate can be driven by visible light for photochemical oxidation [21], while H_2_O_2_ can hardly be activated [22]. Additionally, as a typical organic pollutant, MB is stable under visible light irradiation if no photocatalysts are involved. Therefore, the degradation of MB molecules was attributed to the synergetic effect of Cu_2_ZnSnS_4_ nanoparticle and H_2_O_2_. The H_2_O_2_ enhanced the photocatalytic ability through an efficient charge transfer of the photogenerated carriers from the surface of the Cu_2_ZnSnS_4_ nanoparticle to the MB molecule. Figure 4b,d illustrates the curves of *C*/*C*_0_, where *C*_0_ is the initial concentration of MB and *C* is the concentration of MB at time *t*. Insets in Figure 4b,d present the curve of the corresponding ln(*C*_0_/*C*) versus irradiation time. No linear relationship between irradiation time and ln(*C*_0_/*C*) can be observed when employing the Cu_2_ZnSnS_4_ as a photocatalyst solely. However, with the assistance of H_2_O_2_, a linear relationship between irradiation time and ln(*C*_0_/*C*) is well established, suggesting that the photodegradation of MB over the Cu_2_ZnSnS_4_ nanoparticle and H_2_O_2_ proceeded through the pseudo-first-order kinetic reaction [23]. The first-order reaction rate constant *k*_*1*_ was 0.04 min^−1^, which is comparable to that of TiO_2_-C hybrid aerogel photocatalysts (0.01 ~ 0.06 min^−1^) toward the degradation of MB driven by UV light irradiation [24].

**Figure 5:**
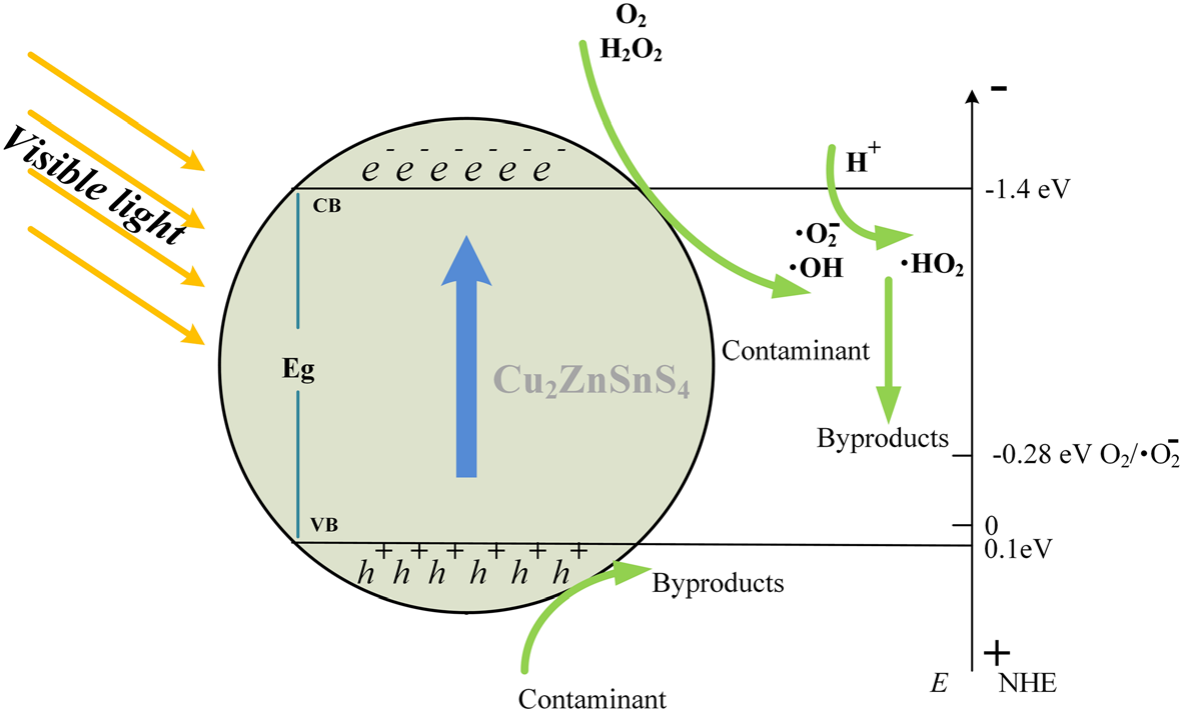
**Schematic diagram for charge carrier separation and photocatalytic contaminant decomposition on Cu**_**2**_**ZnSnS**_**4 **_**photocatalyst under visible light irradiation.**

According to the experiment results and previous reports, the photocatalytic degradation mechanism by the Cu_2_ZnSnS_4_ nanoparticle under visible light irradiation was illustrated in Figure 5 and proposed as follows:

(1) $${\mathrm{Cu}}_{2}{\mathrm{ZnSnS}}_{4}\overset{\mathrm{visible}\;\mathrm{light}}{\to }{\mathrm{Cu}}_{2}{\mathrm{ZnSnS}}_{4}\left(e_{\mathrm{cb}}^{-}+h_{\mathrm{vb}}^{+}\right)$$

When the photogenerated carriers emigrate to the surface of the Cu_2_ZnSnS_4_ nanoparticle, the generated Cu_2_ZnSnS_4_$\;\left(h_{\mathrm{vb}}^{+}\right)\;$provides holes, decomposing the absorbed contaminant.

(2) $${\mathrm{Cu}}_{2}{\mathrm{ZnSnS}}_{4}\left(h_{\mathrm{vb}}^{+}\right)\;+\;\mathrm{contaminant}\to \mathrm{degraded}\;\mathrm{products}$$

However, due to the top of the valence band of Cu_2_ZnSnS_4_ (+0.1 eV versus NHE) is lower than those of · OH/H_**2**_O (+2.27 eV) and · OH/OH^−^ (+1.99 eV), the Cu_2_ZnSnS_4_ nanoparticle cannot further degrade the MB with increasing the irradiation time. More recently, Yu *et al.*[25] have reported that a noble metal such as Au or Pt can greatly improve the photocatalytic ability of the Cu_2_ZnSnS_4_ nanoparticle. The enhancement is attributed to surface plasmon resonance (SPR) effect, which can notably reduce the carrier recombination rate on the surface of the Cu_2_ZnSnS_4_ nanoparticle. In our work, with the addition of a small amount of H_2_O_2_, the generated Cu_2_ZnSnS_4_$\left(e_{\mathrm{cb}}^{‒}\right)$ can react with the added H_2_O_2_, generating · OH, and subsequently photodegrade the absorbed contaminant.

(3) $$H_{2}O_{2}\left(\mathrm{added}\right)+{\mathrm{Cu}}_{2}{\mathrm{ZnSnS}}_{4}\left(e_{\mathrm{cb}}^{-}\right)\to \cdot \mathrm{OH}+{\mathrm{OH}}^{-}$$

(4) ·OH+contaminant→degradedproducts

H_2_O_2_, as an efficient electron scavenger and a source of · OH with high oxidizing ability, was added here to assist the photodegradation of the contaminant. However, an excess amount of H_2_O_2_ will decrease the photocatalytic activity [26]. As shown in Equation 5, the excess H_2_O_2_ scavenges the beneficial OH generating a much weaker hyperoxyl radical of ${\mathrm{HO}}_{2}^{•}$.

(5) $$H_{2}O_{2}+\cdot \mathrm{OH}\to {\mathrm{HO}}_{2}^{•}+H_{2}O$$

Besides, the ${\mathrm{HO}}_{2}^{•}$ will further react with the remaining · OH forming ineffective oxygen and water.

(6) $${\mathrm{HO}}_{2}^{•}+\cdot O\to H_{2}O+O_{2}$$

## Conclusions

In summary, the Cu_2_ZnSnS_4_ nanoparticle was successfully synthesized by a facile microwave fabrication method. The prepared Cu_2_ZnSnS_4_ nanoparticle exhibited a polycrystalline structure with an average diameter of approximately 31 nm. The photocatalytic performance of the Cu_2_ZnSnS_4_ nanoparticle toward degradation of MB in aqueous solution was also investigated. Due to the top of the valence band of Cu_2_ZnSnS_4_, pure Cu_2_ZnSnS_4_ nanoparticle showed a poor visible light photocatalytic ability. However, when employing a small amount of H_2_O_2_ as electron scavenger, the photocatalytic performance was greatly enhanced. The first-order reaction rate constant *k*_*1*_ toward the degradation MB reached as high as 0.04 min^−1^. The synergetic effect between the Cu_2_ZnSnS_4_ nanoparticle and H_2_O_2_ was a key to promote the photodegradation efficiency.

## Competing interests

The authors declare that they have no competing interests.

## Authors’ contributions

ZHZ formulated the idea of investigation and drafted the manuscript. PAZ prepared the Cu_2_ZnSnS_4_ nanoparticles. YLL and AE have taken part in acquisition and interpretation of data. HNJ, JW, and HDL directed the research and made corrections to the manuscript. ZMW supervised the study. All authors have read and approved the final manuscript.

## References

[B1] Liao J Lin S Zhang L Pan N Cao X Li J Photocatalytic degradation of methyl orange using a TiO_2_/Ti mesh electrode with 3D nanotube arrays ACS Appl Mater Interfaces 2011 4 171 177 22117568 10.1021/am201220e

[B2] Ding D Long M Cai W Wu Y Wu D Chen C In-situ synthesis of photocatalytic CuAl_2_O_4_-Cu hybrid nanorod arrays Chem Commun 2009 24 3588 3590 10.1039/b903865e 19521617

[B3] Chandrappa KG Venkatesha TV Sharifah BAH Electrochemical generation of cubic shaped nano Zn_2_SnO_4_ photocatalysts Nano Micro Letters 2013 5 101 110

[B4] Hsiao Y-C Wu T-F Wang Y-S Hu C-C Huang C Evaluating the sensitizing effect on the photocatalytic decoloration of dyes using anatase-TiO_2_ Appl Catal B Environ 2014 148–149 250 257

[B5] De Trizio L Buonsanti R Schimpf AM Llordes A Gamelin DR Simonutti R Milliron DJ Nb-doped colloidal TiO_2_ nanocrystals with tunable infrared absorption Chem Mater 2013 25 3383 3390 10.1021/cm402396c

[B6] Zhang Z Luo Z Yang Z Zhang S Zhang Y Zhou Y Wang X Fu X Band-gap tuning of N-doped TiO_2_ photocatalysts for visible-light-driven selective oxidation of alcohols to aldehydes in water RSC Advances 2013 3 7215 7218 10.1039/c3ra40518d

[B7] Chen C Cai W Long M Zhou B Wu Y Wu D Feng Y Synthesis of visible-light responsive graphene oxide/TiO_2_ composites with p/n heterojunction ACS Nano 2010 4 6425 6432 10.1021/nn102130m 20945928

[B8] Zhang J Chen G Chaker M Rosei F Ma D Gold nanoparticle decorated ceria nanotubes with significantly high catalytic activity for the reduction of nitrophenol and mechanism study Appl Catal B Environ 2013 132–133 107 115

[B9] Gai Y Li J Li S-S Xia J-B Wei S-H Design of narrow-gap TiO_2_: a passivated codoping approach for enhanced photoelectrochemical activity Phys Rev Lett 2009 102 036402 19257373 10.1103/PhysRevLett.102.036402

[B10] Jiang B Zhang P Zhang Y Wu L Li HX Zhang DQ Li GS Self-assembled 3D architectures of Bi_2_TiO_4_F_2_ as a new durable visible-light photocatalyst Nanoscale 2012 4 455 460 10.1039/c1nr11331c 22095258

[B11] Sajjad S Leghari SAK Zhang JL Nonstoichiometric Bi_2_O_3_: efficient visible light photocatalyst Rsc Advances 2013 3 1363 1367 10.1039/c2ra22239f

[B12] Wu W Liang S Chen Y Shen L Yuan R Wu L Mechanism and improvement of the visible light photocatalysis of organic pollutants over microcrystalline AgNbO_3_ prepared by a sol–gel method Mater Res Bull 2013 48 1618 1626 10.1016/j.materresbull.2013.01.011

[B13] Zhu MS Chen PL Liu MH Graphene oxide enwrapped Ag/AgX (X = Br, Cl) nanocomposite as a highly efficient visible-light plasmonic photocatalyst ACS Nano 2011 5 4529 4536 10.1021/nn200088x 21524132

[B14] Chen S Walsh A Gong X-G Wei S-H Classification of lattice defects in the kesterite Cu_2_ZnSnS_4_ and Cu_2_ZnSnSe_4_ earth-abundant solar cell absorbers Adv Mater 2013 25 1522 1539 10.1002/adma.201203146 23401176

[B15] Xinkun W Wei L Shuying C Yunfeng L Hongjie J Photoelectric properties of Cu_2_ZnSnS_4_ thin films deposited by thermal evaporation J Semicond 2012 33 022002 10.1088/1674-4926/33/2/022002

[B16] Wang L Wang W Sun S A simple template-free synthesis of ultrathin Cu_2_ZnSnS_4_ nanosheets for highly stable photocatalytic H_2_ evolution J Mater Chem 2012 22 6553 6555 10.1039/c2jm16515e

[B17] Fernandes PA Salome PMP da Cunha AF Growth and Raman scattering characterization of Cu_2_ZnSnS_4_ thin films Thin Solid Films 2009 517 2519 2523 10.1016/j.tsf.2008.11.031

[B18] Fernandes PA Salomé PMP da Cunha AF Study of polycrystalline Cu_2_ZnSnS_4_ films by Raman scattering J Alloy Compd 2011 509 7600 7606 10.1016/j.jallcom.2011.04.097

[B19] Zou C Zhang LJ Lin DS Yang Y Li Q Xu XJ Chen X Huang SM Facile synthesis of Cu_2_ZnSnS_4_ nanocrystals Crystengcomm 2011 13 3310 3313 10.1039/c0ce00631a

[B20] Serrano J Cantarero A Cardona M Garro N Lauck R Tallman RE Ritter TM Weinstein BA Raman scattering in beta -ZnS Phys Rev B 2004 69 014301

[B21] Zhou G Sun H Wang S Ming Ang H Tadé MO Titanate supported cobalt catalysts for photochemical oxidation of phenol under visible light irradiations Sep Purif Technol 2011 80 626 634 10.1016/j.seppur.2011.06.021

[B22] Sun H Liu S Liu S Wang S A comparative study of reduced graphene oxide modified TiO_2_, ZnO and Ta_2_O_5_ in visible light photocatalytic/photochemical oxidation of methylene blue Appl Catal B Environ 2014 146 162 168

[B23] Mohamed MM Al-Esaimi MM Characterization, adsorption and photocatalytic activity of vanadium-doped TiO_2_ and sulfated TiO_2_ (rutile) catalysts: degradation of methylene blue dye J Mol Catal A Chem 2006 255 53 61 10.1016/j.molcata.2006.03.071

[B24] Shao X Lu W Zhang R Pan F Enhanced photocatalytic activity of TiO_2_-C hybrid aerogels for methylene blue degradation Sci Rep 2013 3 3018 24145581 10.1038/srep03018 PMC3804859

[B25] Yu X Shavel A An X Luo Z Ibáñez M Cabot A Cu_2_ZnSnS_4_-Pt and Cu_2_ZnSnS_4_-Au heterostructured nanoparticles for photocatalytic water splitting and pollutant degradation J Am Chem Soc 2014 136 9236 9239 10.1021/ja502076b 24946131

[B26] Shang M Wang W Sun S Ren J Zhou L Zhang L Efficient visible light-induced photocatalytic degradation of contaminant by spindle-like PANI/BiVO_4_ J Phys Chem C 2009 113 20228 20233 10.1021/jp9067729

